# Complete Genome Classification System of *Rotavirus alphagastroenteritidis*: An Updated Analysis

**DOI:** 10.3390/v17020211

**Published:** 2025-01-31

**Authors:** Ricardo Gabriel Díaz Alarcón, Karina Salvatierra, Emiliano Gómez Quintero, Domingo Javier Liotta, Viviana Parreño, Samuel Orlando Miño

**Affiliations:** 1 Laboratory of Applied Molecular Biology (LaBiMAp), Faculty of Exacts, Chemical and Natural Sciences, National University of Misiones (UNaM), Posadas CP3300, Misiones, Argentina; licengenetica@gmail.com (R.G.D.A.); elgq_11@hotmail.com.ar (E.G.Q.); javierliotta@gmail.com (D.J.L.); 2 National Council for Scientific and Technical Research (CONICET), Av. Mariano Moreno 1375, Lab 105, Posadas CP3300, Misiones, Argentina; 3 Laboratory “MADAR”, National University of Misiones (UNaM), Ruta 12, Km 7 y ½, Posadas CP3300, Misiones, Argentina; kariales@gmail.com; 4 National Institute of Tropical Medicine (INMeT)—ANLIS “Dr. Carlos Malbrán”, Puerto Iguazú CP3370, Misiones, Argentina; 5 National Institute of Agricultural Technology (INTA), IncuINTA, De Las Cabañas y De los Reseros s/n, Hurlingham CP1816, Buenos Aires, Argentina; parreno.viviana@inta.gob.ar; 6 National Institute of Agricultural Technology (INTA), EEA Cerro Azul, National Route 14, Km 836, Cerro Azul CP3313, Misiones, Argentina

**Keywords:** RVA strain classification, strain reference, pairwise distance comparison, phylogeny

## Abstract

*Rotavirus alphagastroenteritidis* is the major causative agent of acute gastroenteritis in both children under the age of 5 and young mammals and birds globally. RVAs are non-enveloped viruses with a genome comprising 11 double-stranded RNA segments. In 2008, the Rotavirus Classification Working Group pioneered a comprehensive and complete RVA genome classification system, establishing a specific threshold, which measures the genetic distances between homologous genes. The aim of this study was to perform an updated systematic analysis of the genetic variability across all RVA genes. Our investigation involved assessing the established cutoff values for each RVA genome segment and determining the need for any updates. To achieve this objective, multiple sequence alignments were constructed for all 11 genes and one for each genotype with discrepancies. Also, pairwise distances along with their cutoff values were evaluated. The analyses provided insights into the current relevance of cutoff values, which remain applicable for the majority of genotypes. In conclusion, this study fortifies the current classification system by highlighting its robustness and accurate genotyping of *Rotavirus alphagastroenteritidis*.

## 1. Introduction

*Rotavirus alphagastroenteritidis* (RVA) is a major pathogen associated with acute gastroenteritis in children, young mammals, and birds worldwide [1]. RVA has a segmented double-stranded RNA genome which consists of 11 genome segments enclosed in a triple-layered icosahedral capsid [2]. The 11 genome segments encode six viral structural proteins (VP1 to VP4, VP6, and VP7) and six nonstructural proteins (NSP1 to NSP6) [3]. Each genome segment, with the exception of gene 11 that encodes two proteins (NSP5 and NSP6), codes for a single viral protein [4]. The inner layer of the rotavirus virion is mainly composed of VP2, which encases VP1; the viral RNA-dependent RNA polymerase, VP3; the viral capping enzyme; and the viral genome [2]. The middle layer of the virion comprises VP6 trimmers exclusively; VP7 and spikes of VP4 compose the outer layer [2]. VP7 and VP4 were the initial basis of a binary classification system defining the G types (glycoprotein) and P types (protease sensitive), respectively. VP7 and VP4 are capable of independently eliciting neutralizing antibodies, initially used to define rotavirus G and P serotypes [4].

For human strains, three genogroups have been established as follows: two major genogroups represented by the reference Wa and DS-1 strains and one minor genogroup represented by reference AU-1 strain [5]. These genogroups were historically determined by hybridization techniques [5], and they were later confirmed by sequencing, establishing particular “genotype constellations” in line with these genogroups. Sequencing rotavirus genomes and phylogenetic and phylodynamic analyses are critical for understanding the patterns of virus evolution. One method that is frequently used to study the genetic distances between virus strains consists of pairwise sequence identity comparisons [6]. Studying evolutionary patterns, the main generator of diversity in rotavirus appears to be point mutations. These occur continuously due to the high error rate of the RVA polymerase [7]. In addition, viral genome reassortments occur between co-infecting strains, often involving zoonotic transmission [1]. During reassortment events certain RVA genes apparently co-segregate, suggesting an important degree of gene linkage [8]. Furthermore, when host restriction has been observed, only RVAs of particular gene constellations can efficiently infect certain hosts. In fact, a detailed look at RVA genome constellations shows a restricted gene pattern in different animal species, with only a few promiscuous genotypes able to infect multiple species [9,10,11].

In the past decades, specific rotavirus strains were associated with specific animal species; however, after the implementation of the new classification system, the host species’ descriptions were improved [9]. Human RVA strains that possess genes commonly found in animal rotaviruses have been isolated from infected children [9]. Strains such as G3 (found commonly in species such as cats, dogs, monkeys, pigs, mice, rabbits, and horses), G5 (pigs), G6 and G8 (cattle), G9 (pigs), and G10 (cattle) have been isolated from the human population throughout the world [9,12,13,14]. On the other hand, the most common RVA genotypes circulating in humans worldwide are G1P[8], G2P[4], G3P[8], G4P[8], G9P[8], and G12P[8] [15]. In a recent review of RVA distribution in animals, it was observed that reassortment occurs frequently. Many genotypes combine constantly and new reassortants continue to appear [9]. These new reassortants may be transferred to other species, leading to unpredictable outcomes and opening the possibility for the emergence of new virulent variants with unforeseen impacts [9].

Nucleotide composition is the simplest way of characterizing genomes [16], and it is essential to the study of viral evolution, particularly the interplay between viruses and host cells [17]. On the other hand, mutation rate estimates vary considerably, even for the same virus [18,19]. Since viral mutation rates have implications for epidemiological surveillance, pathogenesis [20,21], vaccine development [22,23] antiviral therapy [24], and disease management [25,26], it is important to have accurate data at molecular level [27].

To properly study the evolution of rotaviruses, the establishment of a classification system in which individual genes fall into defined clusters/genotypes based on reliable percentage identity cutoff values is crucial [6]. In recent decades, multiple rotavirus strains have been analyzed and compared to one another by the partial or complete sequencing of all 11 gene segments as this approach has allowed researchers to determine direct genetic relationships [6]. The introduction of a new classification system and the creation of the Rotavirus Classification Working Group (RCWG) marked a significant milestone. This system relied on the nucleotide sequence identity cutoff percentages of each of the 11 RVA genome segments [28]. Genetic identity refers to the degree of similarity between two genetic sequences.

To denote the different encoding genes, the proposed notation was employed (Table 1), providing a valuable tool for studying complete rotavirus genomes, also known as genome constellations, that infect various animal species and humans. This approach greatly enhanced our understanding of host restriction, interspecies transmission events, the emergence of reassortant strains post-vaccination, and the overall evolution of RVA.

The identity cutoff percentages were initially determined based on all available strains in 2008. However, since then, a multitude of new strains has been sequenced, leading to the discovery of new genotypes (Table 1). These genotypes have a reference, usually the first reported. The reference strains were listed by Matthijnssens et al. in 2008 [28] and then the RCWG continued reporting new reference strains in their webpage [29]. The updated list of the reference strains is presented in Table 2.

In this study, our objective was to explore the rigor of the classification system for all 11 RVA genome segments by conducting phylogenetic analyses and constructing pairwise sequence identity profiles following the RCWG recommendations. More specifically, we aimed to test the current classification system using the cutoff values established in 2008.

## 2. Materials and Methods

### 2.1. Matrix Construction and Alignment

In February 2020, we obtained nucleotide sequences of RVA genes from the National Center for Biotechnology Information (www.ncbi.nlm.nih.gov/genbank/ accessed on 28 February 2020). The search was conducted using the words “rotavirus A” and “VP7 gene”, for example, to download all the VP7 strains sequences available, and we then used the search terms “rotavirus A” and “VP4 gene”, to download all the VP4 strain sequences available, and so on. To ensure the integrity of our dataset, we made a “depuration process”, which means we meticulously manually filtered out partial sequences lacking a complete open reading frame (ORF), as well as those containing repeated sequences and uncertainties such as ambiguous nucleotides. In instances where only limited data (only a few or even one sequence) were available for specific genotypes, sequences lacking a complete ORF were still included to maintain a comprehensive representation.

To enhance the accuracy and reliability of our analyses, matrix editions (alignments of all sequences, selection of the ORF, etc.) were carried out using AliView v1.26 [22] and Bioedit v7.2.5 [23]. Sequences were meticulously labeled to include genotype, accession number, host, country of origin, and year of collection, providing a detailed context for each sequence.

Subsequently, multiple sequence alignment (MSA) was constructed for each gene using the online server MAFT (https://mafft.cbrc.jp/alignment/server/index.html accessed on 4 March 2020) with default settings. The resulting matrices underwent further refinement and manual editing using AliView and BioEdit to ensure precise alignment. This comprehensive approach to matrix construction and alignment serves as a robust foundation for our subsequent analyses and contributes to the overall reliability of our findings.

This matrix compares the genetic identities between all possible pairs of sequences of a given genotype. Thus, for each genotype, we obtain the distances between all sequences that compound it. In an ideal case, each identity corresponding to a pair of sequences belonging to the same genotype should be greater than the cutoff value, as described in Table 1 for each gene. For the *p*-distance analysis, the criterion we used to consider a single strain to be “out of the cutoff” is that a genetic distance greater than the cutoff value was obtained with any other strain inside that genotype. However, for divergent strains, the decision to classify it as a new genotype should be analyzed by the expert committee. On the other hand, for the K2P analysis we consider the cutoff value only to determine if they are closely related (if the genetic distance is smaller than the cutoff value) or if they are diverging (if the genetic distance is greater than the cutoff value). In other words, the cutoff value in the K2P analysis only involves evolutionary interpretation and is not used for classification purposes.

### 2.2. Genetic Variation

Pairwise genetic distances were analyzed with a *p*-distance model using MegaX software (version 10, 64 bits for Windows) [30] with default settings. To assess genetic similarity, we calculated similarities both within genotypes (intra-genotype) and between genotypes (inter-genotype). These results were used to construct frequency histograms of identities, where the *x*-axis denotes the identities between each pair of sequences, while the *y*-axis represents the number of measured sequence pairs. Additionally, we calculated the best-fitting evolutionary model with the data to construct the phylogenetic trees and determine the pairwise genetic distances, including base and substitutions frequencies (Table A1).

#### 2.2.1. Data Quality: Evolution Model, Nucleotide Frequency, Nucleotide Substitution, and Phylogenetic Information Estimation

Before conducting phylogenetic analyses, a thorough assessment of the dataset information was carried out. The quality analysis revealed key parameters, including the base frequency and nucleotide substitution rate, as outlined in Table A1. The results of this analysis guided the selection of the evolutionary model, with the General Time Reversible (GTR) model featuring the empirical base frequencies (+F) and the reversibility (R) identified as the most suitable [25], as detailed in Table A1.

Furthermore, the presence of a phylogenetic signal was systematically assessed using the likelihood mapping method [28], implemented in IQ-Tree [29]. This methodological step ensured the reliability of the dataset for subsequent phylogenetic analyses by confirming the presence of informative signals in the genetic data.

#### 2.2.2. Phylogenetic Analysis

The construction of phylogenetic trees was executed through the maximum likelihood method utilizing IQ-Tree. To enhance the accuracy of our analyses, we selected the models that best fit each of the eleven datasets, as detailed in Table A1. This approach ensured that the chosen models were tailored to the specific characteristics of each dataset, contributing to a more precise representation of evolutionary relationships.

In assessing the robustness of the phylogenetic tree branches, we employed 10,000 ultrafast bootstrap replicates [30]. This statistical support method provides a reliable estimation of branch confidence, offering a thorough and statistically rigorous evaluation of the inferred phylogenetic relationships.

By incorporating these methodologies, our phylogenetic tree construction process not only utilized advanced computational techniques but also ensured that the selected models and statistical support measures were optimized for the unique features of each dataset. This comprehensive approach enhances the reliability and significance of the phylogenetic inferences drawn from the analyses conducted with IQ-Tree.

Finally, the constructed phylogenetic trees were visually presented using Figtree software version v1.4.4 (http://tree.bio.ed.ac.uk/software/figtree/), offering an intuitive and comprehensive visualization of the evolutionary relationships inferred from the dataset. This meticulous approach to dataset assessment and the subsequent phylogenetic analysis contributes to the robustness and reliability of the evolutionary insights derived from the study.

## 3. Results

### 3.1. Classification System

**Figure 1:**
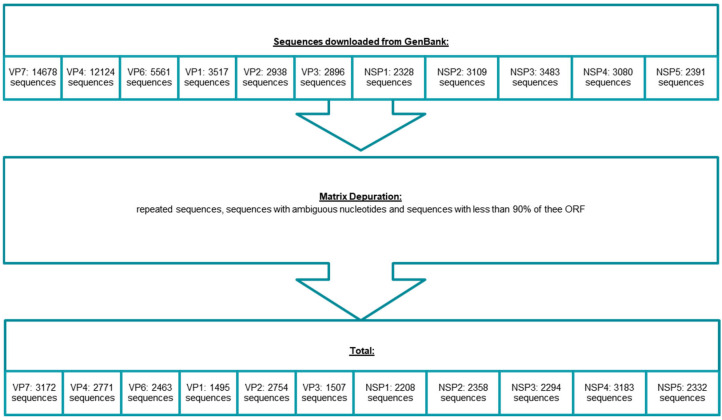
Strain selection criteria and number of strains included in the study.

The full classification analyses were carried out based on a total of 26,537 nucleotide sequences which fulfilled the inclusion criteria, including at least 3172 strains. These sequences were obtained from the GenBank database. We included the nucleotide sequences of VP7 (3172), VP4 (2771), VP6 (2463), VP1 (1495), VP2 (2754), VP3 (1507), NSP1 (2208), NSP2 (2358), NSP3 (2294), NSP4 (3183), and NSP5 (2332) (Figure 1). Table 1 shows the increment of strains between 2008 and the moment this work was performed (February 2020). The column “Current Genotype” indicates the number of genotypes at the moment this paper is written (May 2024); it shows that its number increases quickly. The sum of all genotypes included in this study is 299.

**Figure A1:**
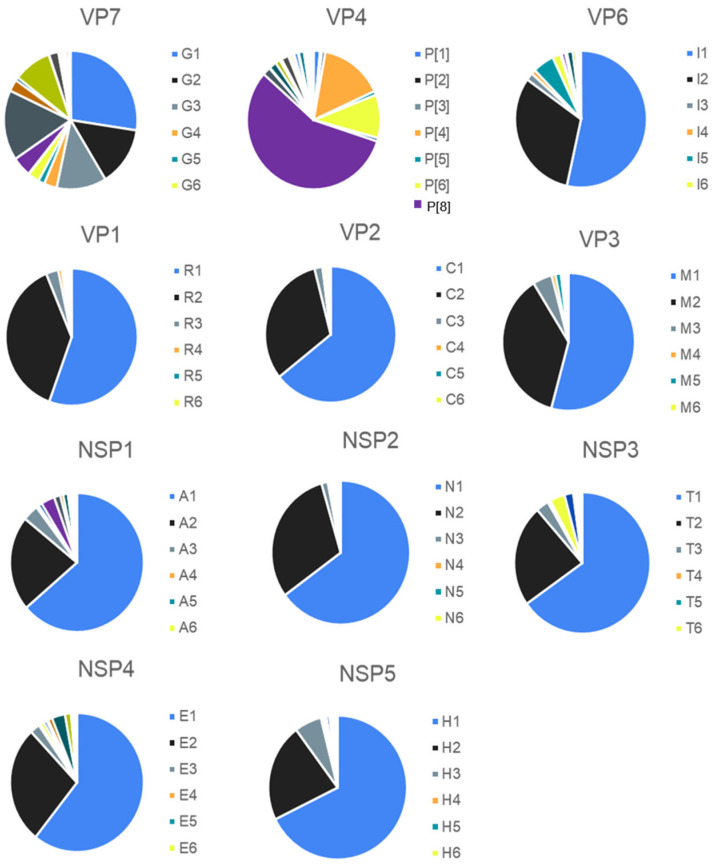
Genotype composition of each gene. This shows that genotypes 1 and 2 are often the most numerous, except for VP7 and VP4.

The relative abundance of genotypes for each gene were also calculated (Figure A1). This figure depicts the dominance of genotypes 1 and 2 over the rest of the genotypes in all genes but VP7 and VP4, where a major diversity can be observed.

The genotyping system was tested with the following two models: the previously established model which uses *p*-distance and is used by the RCWG to classify the new strains, and the model suggested by the software that better fit our dataset (mainly K2P). The histograms of the pair identities were constructed with *p*-distance and the histograms of the pair identities and phylogenetic trees with the K2P model. We will analyze the results separately.

### 3.2. p-Distance Analysis

**Figure 2:**
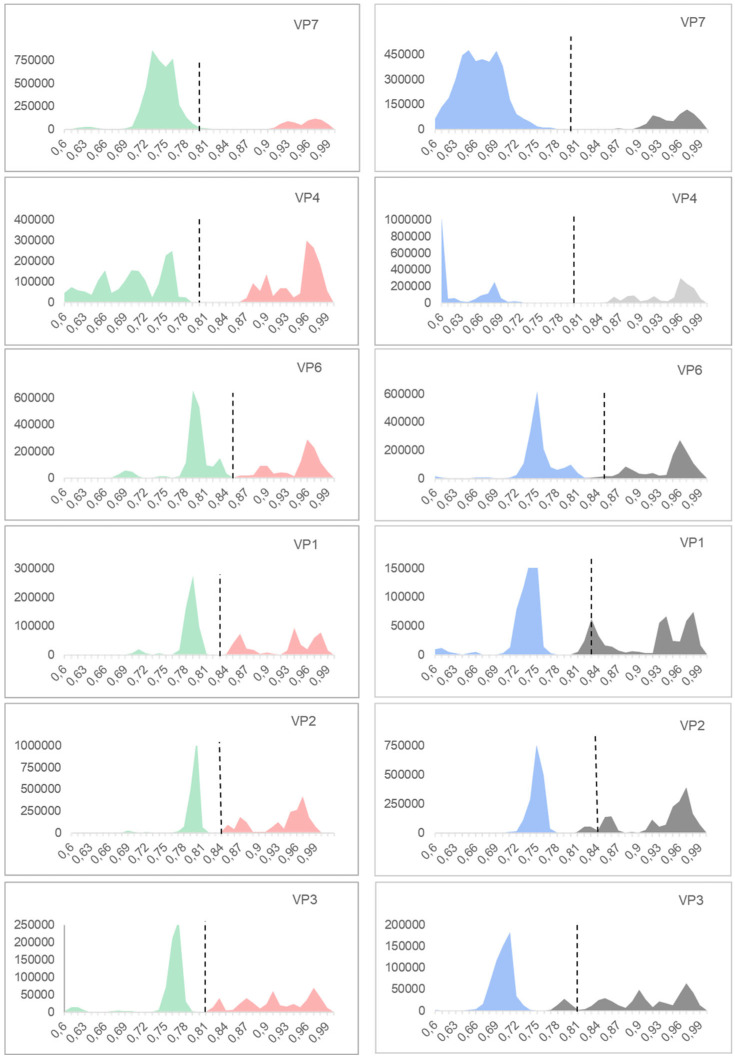
Histograms of pair identities for each Viral Protein (VP). In the ordinates are the identities between each pair of sequences, and the abscissa represents the number of pairs of sequences measured. *p*-distance histograms are on the left; intra-genotype identities are represented in red and inter-genotype identities in green. K2P histograms are on the right; intra-genotype identities are represented in gray and inter-genotype identities in light blue. Dotted lines represent the cutoff value proposed by Matthijnssens et al. in 2008 [28].

**Figure 3:**
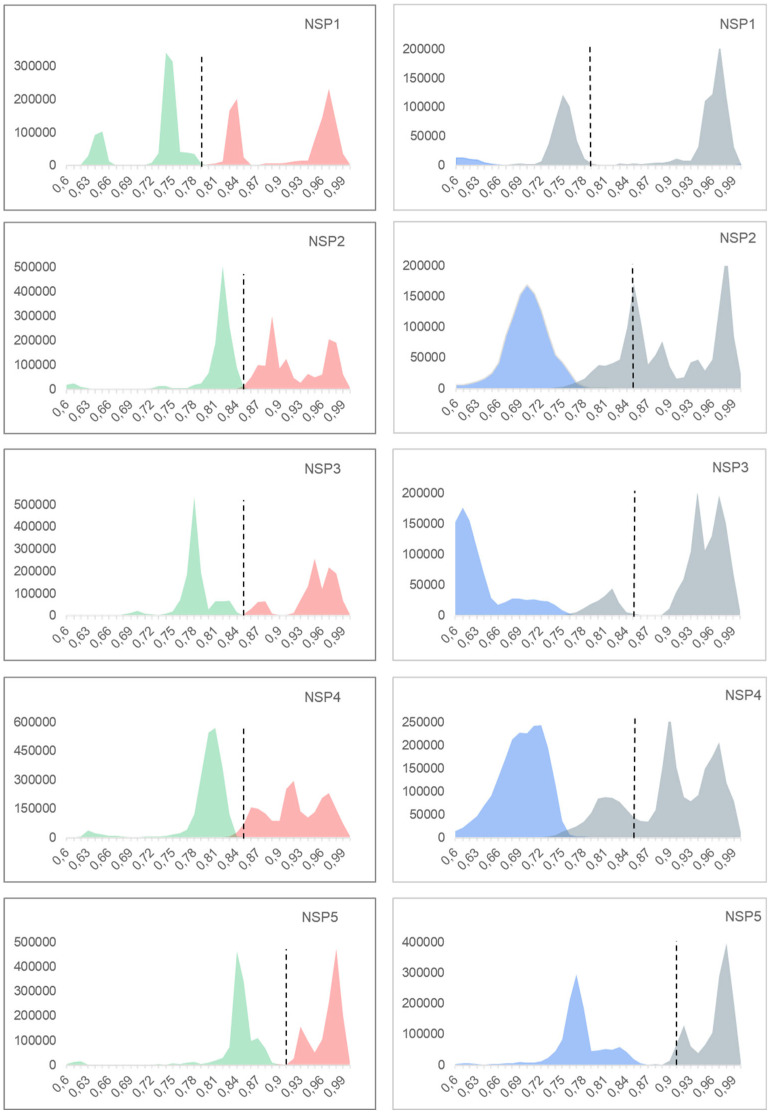
Histograms of the pair identities for each Non-Structural Protein (NSP). In the ordinates are the identities, and in the abscise, the number of pairs of sequences measured are shown. *p*-distance histograms are on the left; intra-genotype identities are represented in red and inter-genotype identities in green. K2P histograms are on the right; intra-genotype identities are represented in gray and inter-genotype identities in light blue. Dotted lines represent the cutoff value proposed by Matthijnssens et al. in 2008 [28].

The *p*-distance histograms reveal a clear differentiation between intra- and inter-genotype distances (Figure 2 and Figure 3). It can be observed that the inter- and intra-genotype distances are clearly differentiated and there is very little overlap. This is evidenced when we observed that the number of strains which falls “outside of the cutoff value” (Table 3) was only 169 for the *p*-distance model. This represents 0.63% (169/26,537) of the total analyzed pairwise identities. Of these, 44.9% (76/169) represents NSP4 outliers. On the other hand, VP7 has only one strain out of the cutoff value (D86277) corresponding to the G3 genotype. Meanwhile, P[8] has 10 strains out of the cutoff value (representing 0.64% of the strains included in these genotypes). The P[15] genotype has only one strain out of the cutoff value and VP1, VP3, and NSP1 have no strains outside of the cutoff. On the other hand, for NSP4, the E3 genotype has 19.5% (15/77) strains out of the cutoff value, and for NSP5 H10 (5/5), H14 (2/2), and H15 (2/2), 100% of the strains are out of the cutoff value, although only a few strains were analyzed for these genotypes.

### 3.3. K2P Analysis: Outer Capsid Proteins (VP7, VP4, and VP6)

**Figure A2:**
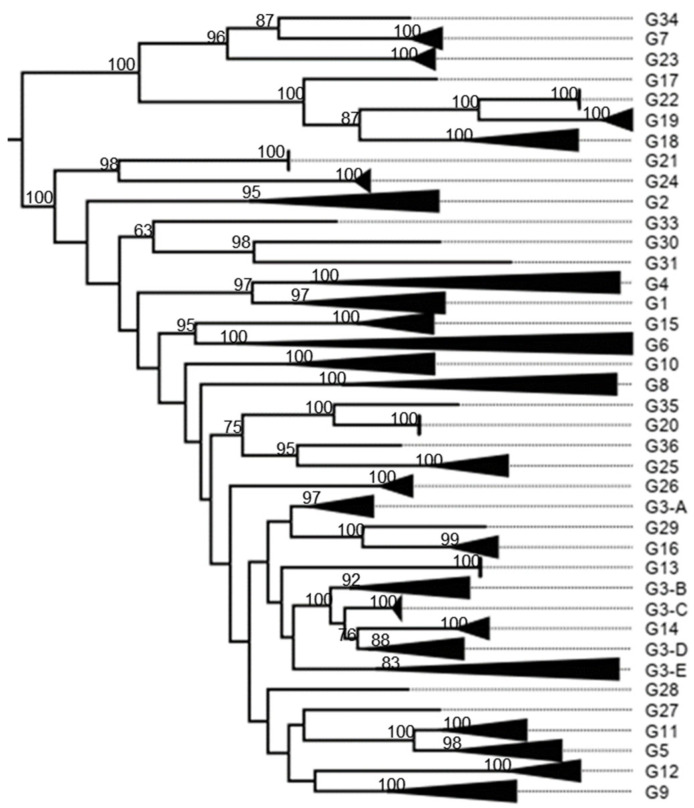
VP7 middle-point rooted phylogenetic tree. G3 shows two principal branches, G3-A with close phylogenetic relationships with G16 and G29 and G3-B related to G13 and G14.

Among the genotypes described for VP7, 89% (32/36) of the genotypes showed that their strains are closely related according to our analysis. Moreover, 11% (4/36 genotypes) exhibited strains with identities ranging from 0.80 to 0.62. These genotypes were G3 with 17% (66/384) of strains, G6 with 73% (64/87), G8 with 42% (63/148), and G10 with 22% (20/88). All genotypes, except for G3, maintain a monophyletic origin within the phylogenetic tree (Figure A2).

**Figure A3:**
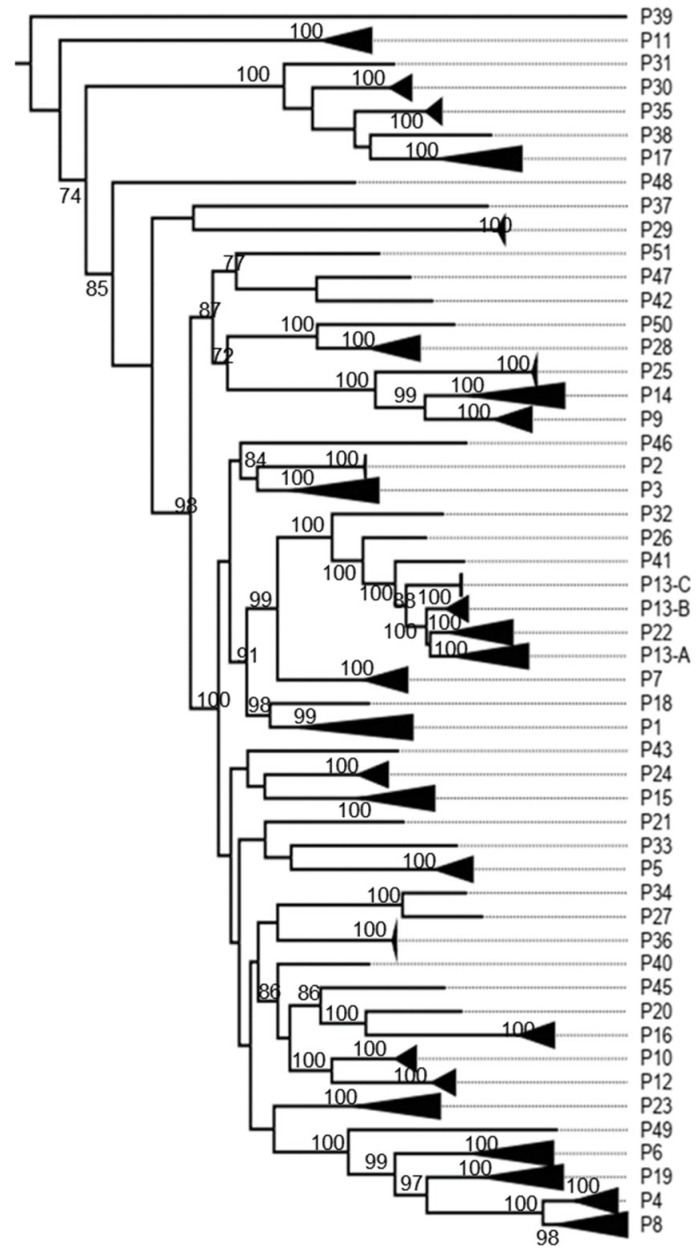
VP4 middle-point rooted phylogenetic tree. P[13] shows close relationships with P[22] and P[41].

Among the genotypes described for VP4, 92% (47/51 genotypes) showed a consistent distance according to our analysis (Figure 2). However, 8% (4/51 genotypes) exhibited strains with identities ranging from 0.79 to 0.73. These genotypes were P[1] with 26% (9/34) of the strains, P[3] with 83% (20/24), P[13] with 35% (5/14), and P[14] with 12% (6/49). Evolutionary relationships between groups are shown with a middle-point root of VP4, where all groups are clearly defined (Figure A3). P[4], P[6], and P[8], all described in humans, are grouped together on the tree and the rest of the genotypes represent a heterogeneous group with a broad range of hosts. Concerning P[13], Figure A3 shows that it is a polyphyletic group. The strains mainly correspond to oriental porcine strains, but one human strain from Belgium and one porcine strain from Spain were also reported.

**Figure A4:**
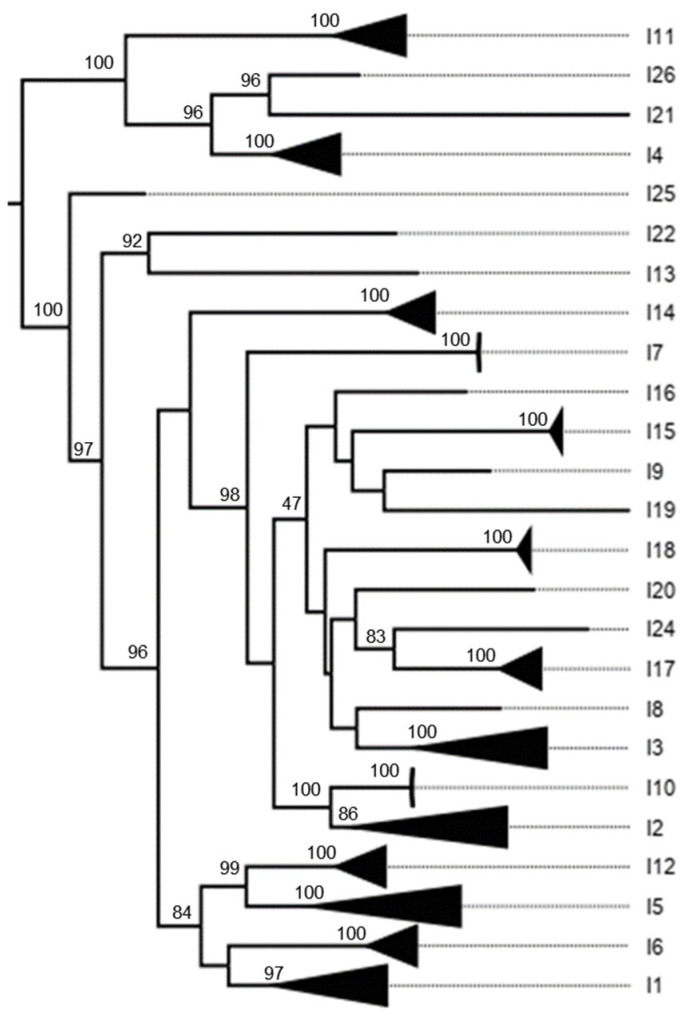
VP6 middle-point rooted phylogenetic tree.

Among the genotypes described for VP6, 88% (23/26) showed a consistent distance according to our analysis (Figure 2). Only 12% (3 out of 26 genotypes) exhibited strains with identities ranging from 0.851 to 0.78. These genotypes were I2 with 9% (72/773) of the strains, I3 with 28% (12/43), and I12 with 67% (11/17), as shown in Figure A1. The VP6 phylogenetic tree with a middle-point root in Figure A4 shows all genotypes have monophyletic origins.

### 3.4. K2P Analysis: Inner Capsid Proteins (VP1, VP2, and VP3)

**Figure A5:**
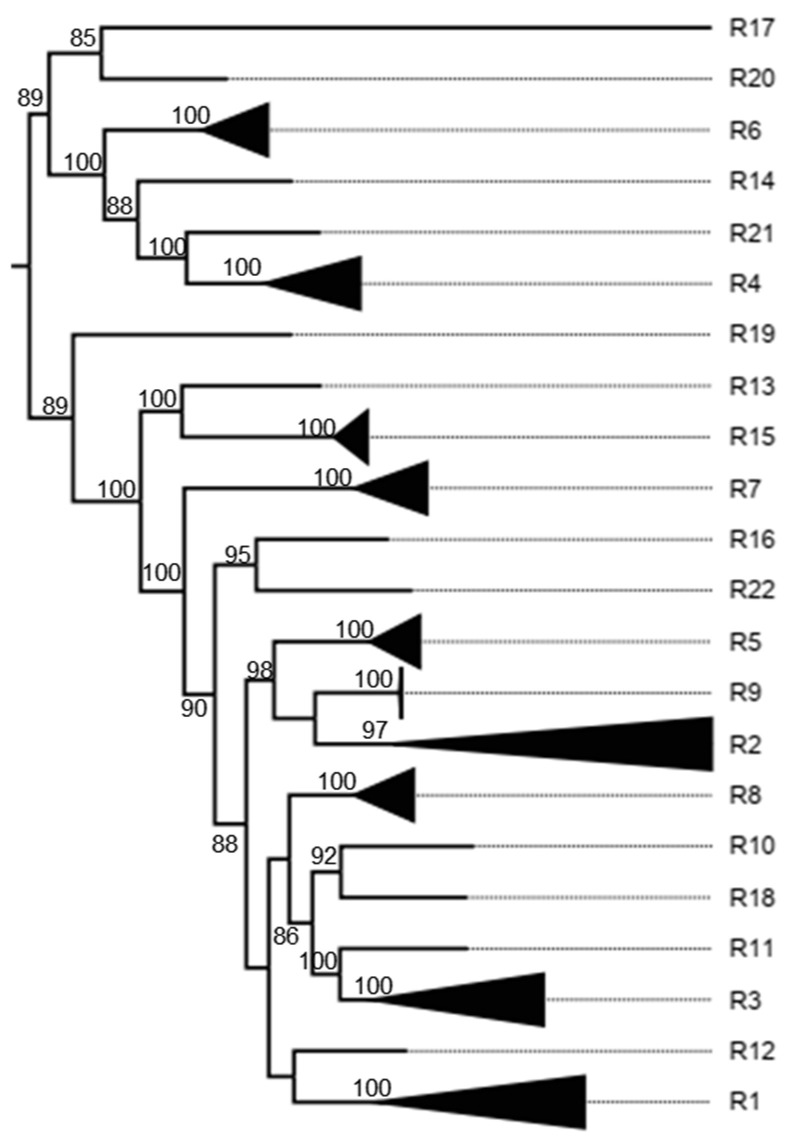
VP1 middle-point rooted phylogenetic tree.

Among the genotypes described for VP1, 87% (19/22 genotypes) showed a consistent distance with the classification method (Figure 2). The other 13% (3/22 genotypes) exhibited strains with low identities ranging from 0.831 to 0.79. These genotypes were R1 with 7% (65/830) of the strains out the cutoff value, R2 with 31% (181/571), and R3 with 35% (16/45). The VP1 phylogenetic tree is shown in Figure A5. Two major groups are observed as follows: R4, R6, R14, R17, R20, and R21 are together in one branch and the rest of the genotypes are in another branch.

**Figure A6:**
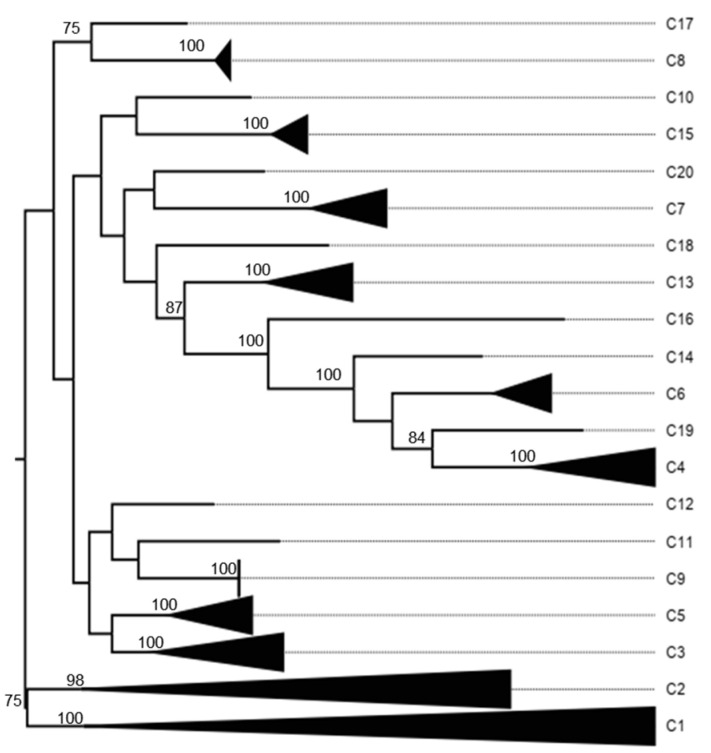
VP2 middle-point rooted phylogenetic tree.

Among the genotypes described for VP2, 90% (18/20) showed a consistent distance according to our analysis (Figure 2). The other 10% (2/20 genotypes) exhibited strains with low identities in a range from 0.841 to 0.69 (Table A1). These genotypes were C2 with 22% (195/868) of the strains and C4 with 33% (4/12). We also observed that strain RVA/Cow-wt/SVN/SI-B17/2004/G6P[11] (access number JX402792) showed distances higher than the cutoff value for most of C2 strains. The VP2 phylogenetic tree showed two branches, one containing C1, C2, C3, C5, C9, C11, and C12 and the other branch containing the rest of the genotypes (Figure A6).

**Figure A7:**
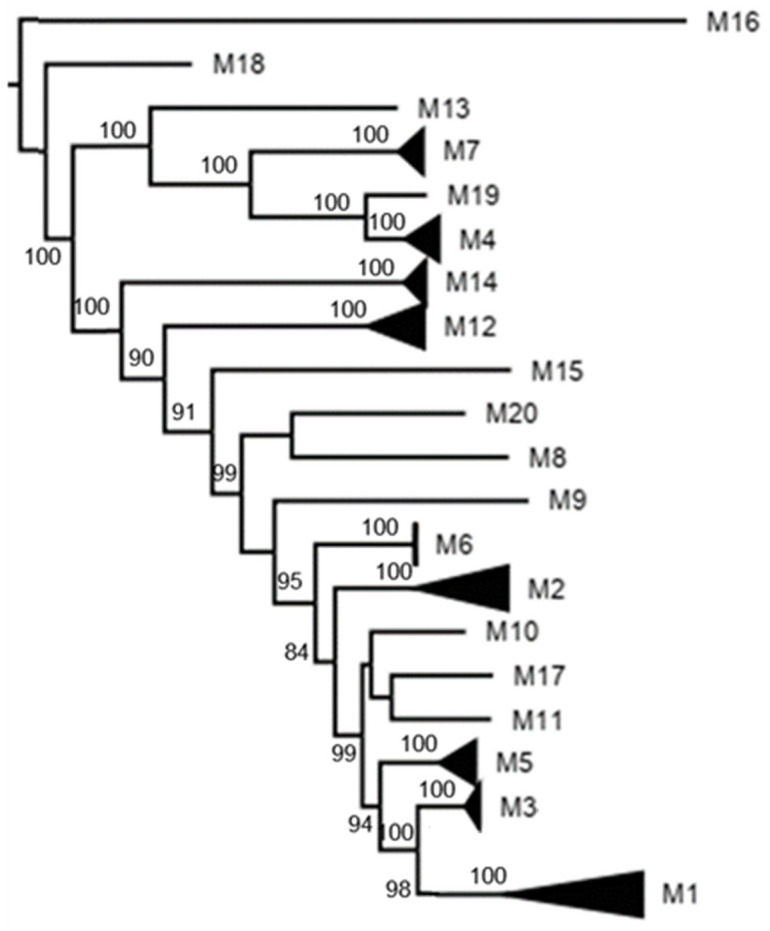
VP3 middle-point rooted phylogenetic tree.

Among the genotypes described for VP3, 90% (18/20 genotypes) showed a consistent distance according to our analysis (Figure 2). The other 10% (2/20 genotypes) exhibited strains with low identities in a range from 0.811 to 0.76 (Table A1). These genotypes were M2 with 25% (137/557) of the strains and M3 with 39% (28/71). The phylogenetic tree shows M16 as a separate distant group from the rest of the genotypes in Figure A7.

### 3.5. Other Model Analysis: Non-Structural Proteins

**Figure A8:**
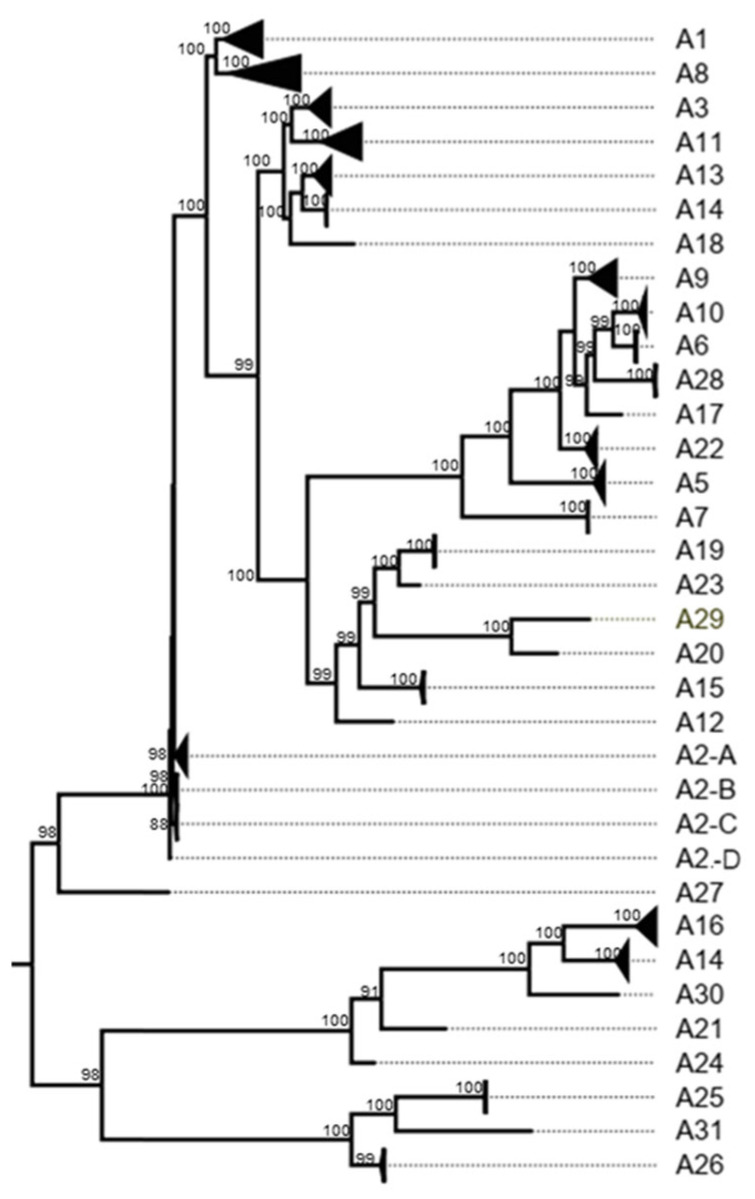
NSP1 middle-point rooted phylogenetic tree.

Among the genotypes described for NSP1, 71% (25/31 genotypes) showed a consistent distance according to our analysis (Figure 3). The other 29% (6/31 genotypes) exhibited strains with low identities in a range from 0.791 to 0.57. These genotypes were A1 with 28% (395/1405) of the strains, A3 with 51% (43/84), A8 with 52% (38/73), A9 with 48% (16/33), A11 with 20% (5/25), and A19 with 100% (6/6). The phylogenetic tree shows six branches. The A27 genotype includes only one sequence, which is far away from the other genotypes (Figure A8).

**Figure A9:**
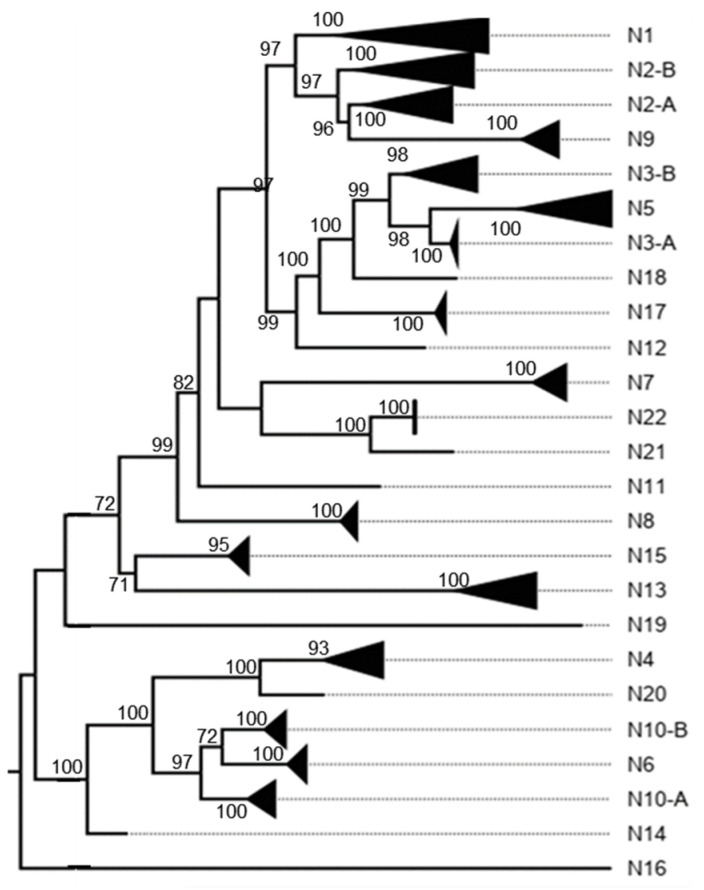
NSP2 middle-point rooted phylogenetic tree. N2 shows a close relationship with N9. N3 shows a close relationship with N5. N10 shows a close relationship with N6.

Among the genotypes described for NSP2, 77% (17/22 genotypes) showed a consistent distance according to our analysis (Figure 3). Only 23% (5/22 genotypes) exhibited strains with low identities ranging from 0.84 to 0.65. These genotypes were N1 with 36% (587/1600) of the strains, N2 with 95% (616/643), N3 with 62% (23/37), N4 with 14% (2/14), and N10 with 75% (6/8). The phylogenetic tree shows that most N-genotypes are monophyletic branches (Figure A9), while N2, N3, and N10 are polyphyletic groups. N2 shows the following two lineages: N2-A and N2-B. The N9 genotype is clustering as a branch within the N2-A lineage. N3 has the following two lineages: N3-A and N3-B, with N5 genotype as a related branch to these two lineages. N10 has the following two lineages: N10-A and N10-B, with N6 as a related branch these two lineages (Figure A9).

**Figure A10:**
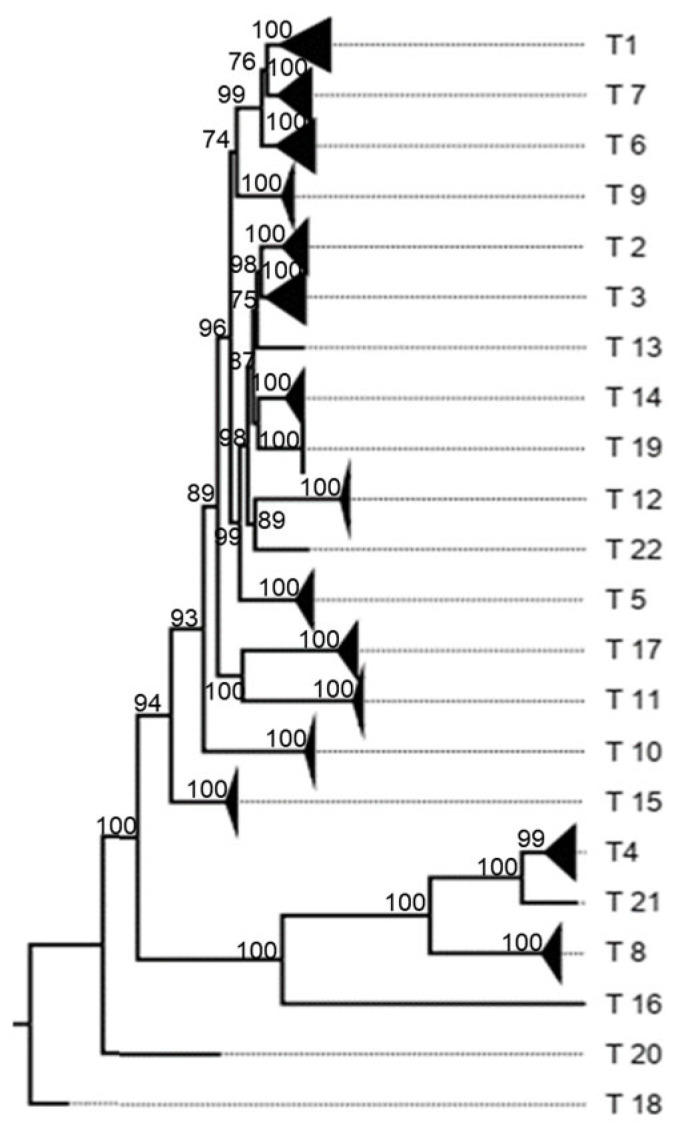
NSP3 middle-point rooted phylogenetic tree.

Among the genotypes described for NSP3, 73% (16/22 genotypes) showed a consistent cutoff value according to our analysis (Figure 3). The 27% (6/22 genotypes) exhibited strains with low identities in a range from 0.851 to 0.66. These genotypes were T1 with 8% (127/1496) of the strains, T3 with 77% (54/70), T4 with 60% (6/10), T6 with 80% (65/81), T7 with 4% (2/48, strains RVA/Pig-wt/ESP/F456/2017/G5P[13] (MH238143) and RVA/dog-tc/CHN/SCCD-A/2017/G9P[23] (MH910071)), and T14 with 60% (3/5). In the phylogenetic tree, T4, T8, T16, and T21 form a branch. On the other hand, T18 were in a branch separate from all other genotypes (Figure A10).

**Figure A11:**
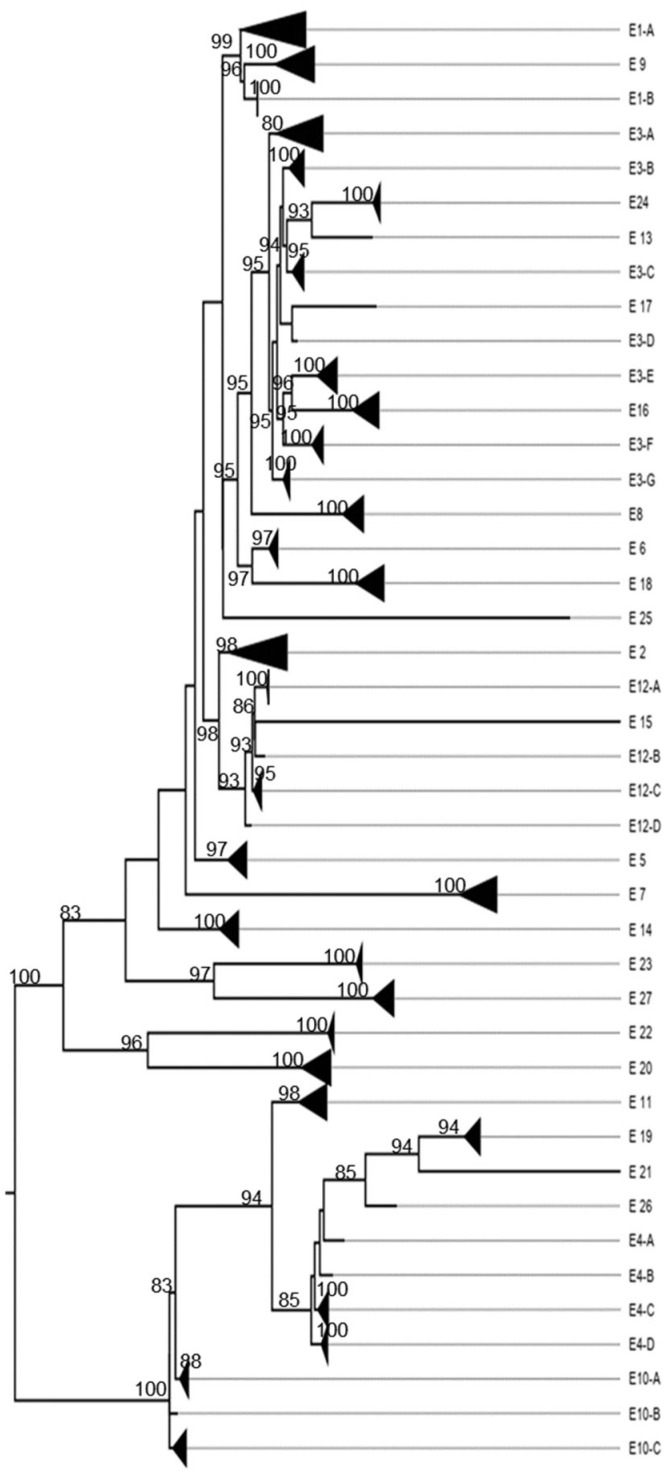
NSP4 middle-point rooted phylogenetic tree. E1 shows a close relationship with E9. E3 shows a close relationship with E13, E16, E17, and E24. E4 shows a close relationship with E19, E21, and E23.

Among the genotypes described for NSP4, 74% (20/27 genotypes) showed a consistent distance according to our analysis (Figure 3). The other 26% (7/27 genotypes) exhibited strains with low identities in a range from 0.851 to 0.63. These genotypes were E1 with 11% (216/1952) of the strains, E2 with 87% (772/881), E3 with 48% (37/76), E4 with 33% (2/6, strains RVA/dove-wt/JPN/PO-13/2001/G18P[17] (AB009627) and RVA/VelvetScoter-tc/JPN/RK1/1989/G18P[17] (LC088105)), E7 with 4% (1/24, strain RVA/mice/USA/EC/1997/GxP[x] (U96337)), E9 with 9% (1/11, strain RVA/Pig-wt/ESP/F438/2017/G5P[19] (MH238162)), and E11 with 1% (1/103, strain RVA/Chicken-tc/xxx/BRS-115/xxx/G7P[35] (KJ725026)). Some other points to mentions are that for NSP4 genotypes which are in the E18 and E20 genotypes, there were only two strains in each genotype, and the distance between them is out of the cutoff value (strains RVA/Rat-wt/ITA/Rat14/2015/G3P[3] (KX398368) and RVA/Rat-wt/GER/KS-11-573/2011/G3P[3] (KJ879457) for E18 and RVA/Bat-wt/CRC/KCR10-93/2010/G20P[47] (MN551607) and RVA/Human-wt/SUR/2014735512/2013/G20P[28] (KX257412) for E20). We also observed that RVA/cat-wt/JPN/FRV70/2001/G3P[3] (AB048196) and RVA/cat-wt/JPN/FRV303/2001/G3P[3] (AB048199), which belong to the E3 genotype, showed low identities across all E3 strains. The phylogenetic tree shows polyphilia in the following strains: between E1 and E9; between E12 and E15; between E4 and E19, E21, and E26; between E3 and E13, E16, E17, and E24 (Figure A11).

**Figure A12:**
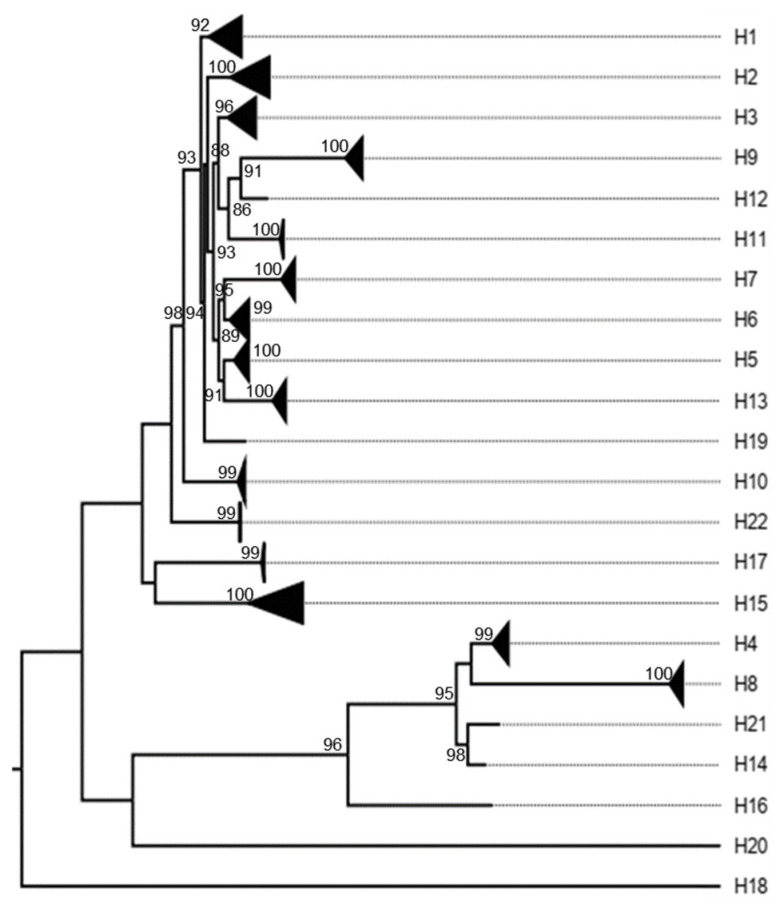
NSP5 middle-point rooted phylogenetic tree.

Among the genotypes described for NSP5, 77% (17/22 genotypes) showed a consistent distance according to our analysis (Figure 3). The other 23% (5/22 genotypes) exhibited strains with low identities in a range from 0.911 to 0.45. These genotypes were H1 with 12% (194/1580) of the strains, H2 with 1% (7/514), H3 with 28% (41/147), H4 with 45% (5/11), H6 with 45% (5/11), and H10 with 100% (5/5). We observed that the H14 and H15 genotypes had only two strains each and showed low identities. We also found that strain KP258408, which belongs to the H3 genotype, showed distances above the cutoff value with all H3 strains. In the phylogenetic tree, we observed one branch which contains H4, H8, H14, H16 and H21, with H18 in a branch separate from the others, and the rest of genotypes in another branch (Figure A12).

### 3.6. Comparison Between the p-Distance and K2P

We compared the performance of the *p*-distance and K2P models. The results are summarized in Table 4. We used the cutoff value established by Matthijnssens et al. in 2008 to determine whether the K2P results were consistent.

## 4. Discussion

In this study, we tested the classification system for all 11 RVA genome segments by constructing pairwise sequence identity profiles using the *p*-distance and K2P models and then conducting phylogenetic analyses using the information available up to 2021. Our objective was to determine whether the current cutoff values remain up to date. With this purpose we selected a dataset of 26,537 strain nucleotide sequences belonging to 299 genotypes obtained from the GenBank database. We found that most genotypes (99.4%) for each gene segment exhibited high consistency in their cutoff value based on the analysis and only a small percentage of genotypes showed strains with identities out of the cutoff value (0.63% of strains).

### 4.1. p-Distance Model vs. K2P

The established genotyping system demonstrated high efficacy in classifying rotavirus strains. A total of 93.4% of the genotypes (20/299) exhibited cutoff values consistent with those analyzed using the *p*-distance model, suggesting that the current classification framework remains applicable for most RVA genes. Additionally, 6.6% of the strains had 99% of their values aligned with the established cutoffs. Table 4 summarizes the performance of the *p*-distance model for classification purposes. This model effectively distinguishes nearly all RVA strains in their respective genotypes, even with the limited sequence information this model uses. All the evidence we found indicates that the *p*-distance model is more suitable for classification compared to other models (K2P), as the classification performance based on the *p*-distance model was superior to that of K2P, as shown in Figure 2 and Figure 3.

### 4.2. K2P Model

The strains analyzed with K2P and the other models indicate the presence of genetic variations, which could have originated from point mutations or other mechanisms [31,32]. The phylogenetic analysis provided additional insights into the evolutionary relationships of the genotypes [6]. In some cases, the phylogenetic trees confirmed the monophyletic nature of the genotypes [31,33,34], while in others, the trees revealed polyphyletic or heterogeneous clustering, suggesting the existence of distinct lineages within those genotypes and genotypes that fall inside other genotypes. This is an interesting effect of the evolutionary process because strains represent living organisms. These organisms are evolving, even as we observe them, and the phylogenetic tree is like a snapshot of their current state. In this snapshot, we can see the direction in which the strains are evolving, where they come from, and where they are heading in evolutionary terms. This explains the presence of lineages within strains, and it is natural for it to be this way. However, we agree that classification is a convention that is not necessarily consistent with evolutionary relationships. It is supported by using the simplest genetic distance model (*p*-distance), which does not include evolutive information for genotype classification but is the model that best discriminates genotypes based on their nucleotide sequence. The best example for this is G3; the phylogenetic analysis of G3 reveals a complex evolutionary structure, with this strain divided into five distinct lineages as follows: A, B, C, D, and E. Adding to this complexity, G13, G14, G16, and G29 are positioned intermediately among these lineages. This intermediate placement suggests overlapping evolutionary relationships, indicating the possible emergence of new strains, probably due to G3 diverging to produce the other strains. These highlight the dynamic nature of strain evolution, where distinct lineages can still share close genetic ties with multiple related strains. The evolutionary relationships between strains P[13] and P[22] are another example, as P[13] is located in a well-supported branch with a bootstrap value of 100. This branch is divided into the following three lineages: A, B, and C. P[22] is positioned between lineages A and B of P[13], indicating that P[22] shares more similarity with this strain than with the rest of the VP4 strains. The placement of P[22] highlights the dynamic nature of these processes and the importance of using phylogenetic trees as snapshots to interpret evolutionary pathways. On the other hand, we have the NSP2 strains. N2 is divided into two lineages, A and B, with N9 positioned between them, suggesting a closer evolutionary relationship with both lineages. Similarly, N3 is also divided into two lineages, A and B, and N5 is located in the middle of these two groups. Finally, N10 is split into two distinct lineages, A and B, with N6 occupying an intermediate position between them. These findings highlight consistent patterns of lineage division and the intermediate placement of related strains, offering further insights into the evolutionary dynamics within these groups. These examples show us the need to study how different genotypes evolve, but these questions are beyond the scope of this work.

Other genotypes phylogenetically closely related are E1 and E9, E12 and E15; E3 and E13, E8, E16, E17 and E24; E4 with E19, E21 and E26. And not only phylogenetically, as in the case of G3 with G14, which are more closely related to each other serologically than different genotypes usually are [35]. Antigenic analysis, as a virus neutralization test, could be carried out in order to response to these questions, at least for VP7 and VP4.

The viral proteins (VPs) and the non-structural proteins (NPSs) will be discussed in order, from simplest to most complex to analyze.

#### 4.2.1. Proteins Forming the Triple Layer Particles

Only three VP6 genotypes demonstrated low identities as follows: I2, I3, and I12. Many studies use phylogeny to identify the genotype of VP6 strains in combination with their pair identities, and high identities were observed [36,37,38,39,40,41,42,43,44].

VP1, VP2, and VP3 show close relationships with the strains belonging to each genotype. The phylogenetic tree shows that no polyphyletic groups are present.

VP7 presents only four genotypes (G3, G6, G8, and G10) with low identities. We identify G3 as a polyphyletic group, in accordance with previous studies [42,44,45]. A molecular analysis to examine the genetic variation within the VP7 of 27 G3 human and animal rotavirus strains was performed more than three decades ago by Nishikawa et al., 1989 and showed an overall sequence identity of 85% or higher [33]. A higher degree of overall VP7 sequence similarity was observed among strains from the same animal species when compared to strains from different animal species, suggesting that these were VP7 species-specific sequences. Moreover, VP7 had serotype-specific regions, where genetic variations identified among strains of different serotypes were highly conserved among G3 from the same species [33]. Furthermore, Nishikawa et al. found that the varying reactivities of anti-VP7 monoclonal antibodies with the 27 strains studied were consistent with the occurrence of antigenic variation among serotype 3 strains. The corresponding phylogenetic tree suggested that G3 rotavirus strains from different animal species were more closely related to each other than to rotavirus strains of different G genotypes [33]. This finding poses the query regarding the classification of these strains. Phylogenetic analysis is a useful supporting tool for the classification of all genotypes except G3, which is the most variable genotype.

Notably, prior research identified three lineages within G5 [46] using 28 strains, while our study, encompassing 48 strains, reaffirms the monophyletic nature of G5. Various studies have explored VP7 genotypes and strains (G6, G5, G3, G8, G9, G12), primarily for classification purposes [36,38,40,43,45,47,48,49]. Interestingly, a previous study tentatively designated some G3 strains exceeding the cutoff value as G16 [6]. However, in our findings, G16 strains were appropriately grouped within the correct distance to G3 strains, suggesting that the discrepancies in previous studies may be due to the limited number of strains analyzed. Additionally, our results revealed that G6 exhibited substantial intra-genotype diversity in prior studies, with five distinct lineages identified. Furthermore, we observed that G15 was classified as a lineage of G6 rather than a separate G-type [39].

Concerning VP4, only four genotypes (P[1], P[3], P[13], and P[14]) possess strains with low identities. These strains share a monophyletic origin within the phylogenetic tree, with the exception of P[13]. Other studies that focused on P[3] [40,44] and P[13] [39,42,45] did not find any subgroups or polyphyletic origin for these genotypes. The exclusion of identities between closely related P[4] and P[8] genotypes—consistent with the methodology employed by Matthijnssens in 2008 [4], who find identities between P[8] and P[4] ranged from 84% to 89%, completely above the 80% cutoff value—reinforces the idea that P[8] and P[4] are not only closely related P genotypes but are also subtypes corresponding to the distinct serotypes P1A and P1B, respectively, which were initially thought to be part of a single P serotype (P1) [50,51,52]. This exclusion significantly influences the accurate classification of new segments. Prior research has extensively investigated P[4] and P[8] genotypes, along with their respective alleles, contributing valuable insights to the understanding of these genomic elements [36,37,40,43,49].

#### 4.2.2. Nonstructural Proteins

NSP5 exhibits the highest cutoff among all RVA genes, which shows that it is the most conserved gene. We found that 5 out of 22 genotypes possess strains with low identities. Specifically, genotypes H1 and H2 exhibited 12% and 1% of their strains, respectively, with high genetic distances. H10 needs further analysis because the identities that exhibit its sequences are lower than 0.78. Previous studies did not find a polyphyletic origin or groups within this genotype [37,38,39,40,42,43,44].

Concerning NSP3, six genotypes (27%) showed low identities. These included T3 with 77% (54/70), T4 with 60% (6/10), T6 with 80% (65/81). Further evolutionary analysis is needed to determine why many strains of this gene showed low identities. Previous studies did not find a polyphyletic origin or groups within this genotype, possibly because the low number of strains used [37,38,39,40,42,43,44].

In the case of NSP1, we found a large peak of inter-genotypes pair identities (between 0.62 and 0.80 in Figure 3). NSP1’s cutoff stands at 0.79, the lowest among all genes, being the most variable of the 11 genes. This gene also needs deeper evolutionary analysis. Previous studies were conducted on NSP1 to compare the local strains with other strains, but no polyphyletic groups were observed [37,38,39,40,42,43,44,49].

Previous studies used the classification system for NSP2 but did not find a polyphyletic origin or groups within this genotype [37,38,39,40,42,43,44]. This could be due to these works having classification purposes, with just a few strains being used.

Concerning NSP4, three genotypes, E1, E2, and E3, comprise numerous strains with low identities. This gene presents high variability and the phylogenetic trees show the following three polyphyletic groups: E3, E10, and E12. This shows that these strains are closely related and the genotypes are changing over time. Previous studies conducted on NSP4 have found that it is a monophyletic group [36,37,38,39,40,41,42,43,44].

Overall, as new strains are sequenced and more genetic data become available, it is important to regularly check and update the classification system used for RVA classification. It is crucial to reassess the cutoff values and refine the classification system to accurately reflect the genetic diversity and evolutionary relationships of the rotavirus strains when needed [6].

Additional investigations are imperative to explore strains with high genetic distances and assess whether these strains are diverging into novel subtypes (lineages). A more in-depth analysis of the evolutionary path is needed for specific strains, such as G3 with G13, G14, G16 and G29; P[13] with P[22]; N2 with N9; N3 with N5; N10 with N6, E1 and E9; E12 and E1; E3 with E13, E8, E16, E17 and E24; E4 with E19, E21 and E26. The rationale behind this inquiry is rooted in the observation that all these genotypes harbor strains with low identities, and the phylogenetic analysis indicates their inclusion within other genotypes. This nuanced exploration will contribute to a deeper understanding of the genetic diversity and relationships between these strains, potentially leading to the identification of novel genotypic distinctions or subtypes.

Additionally, ongoing surveillance and sequencing efforts will continue to contribute to our understanding of rotavirus evolution and the impact of vaccination on strain diversity [6,15,53,54,55]. This study employed a large dataset of rotavirus nucleotide sequences to investigate the genotype diversity and evolutionary relationships of various gene segments. The analysis revealed variations in genotype diversity among different genes, with VP7 and VP4 showing the highest diversity, as expected for the neutralizing antigens of the virus. There is remarkable consistency with the established cutoff values that have served as an excellent tool for RVA classification over the last fifteen years.

## 5. Conclusions

In conclusion, the application of the rotavirus classification system to numerous rotavirus strains (more than 3170 strains and 26,500 sequences) allowed the accurate differentiation of all genotypes within each gene, showing the robustness of the system. Furthermore, the system facilitates the detection of multiple reassortment events and transmissions between species, allowing them to trace the origins of this variability and to identify the parental strains of emerging variants. Our study provides valuable information that promotes the utilization of the rotavirus genotype classification system by testing its robustness. Finally, our phylogenetic analyses show the complexity of the evolutionary relationships within this viral species and urge us to continue studying the evolution of rotaviruses to better understand its dynamic and evolutionary process.

## Figures and Tables

**Table 1:** Information about the encoded proteins of RVA. The classification notations used, the number of sequences analyzed in a previous work (2008), the present work (2024), and the current number of genotypes [29].

Comparison Between Previous and Present Work								
Gene	Encoded Protein	Notation	Cutoff Value	2008 *		This Work (2024)		Current Genotype
				Strains	Genotypes	Strains	Genotypes	
VP7	**G**lycosylated	G	80	1000	15	3172	36	42
VP4	**P**rotease-sensitive	P	80	190	27	2771	51	58
VP6	**I**nner capsid	I	85	142	10	2463	26	32
VP1	**R**NA-polymerase	R	83	58	4	1495	22	28
VP2	**C**ore protein	C	84	58	5	2754	20	24
VP3	**M**ethyltransferase	M	81	67	6	1507	20	23
NSP1	Interferon **A**ntagonist	A	79	100	14	2208	31	39
NSP2	**N**TPase	N	85	71	5	2358	22	28
NSP3	**T**ranslation enhancer	T	85	77	7	2294	22	28
NSP4	**E**nterotoxin	E	85	100	6	3183	27	32
NSP5	p**H**osphoprotein	H	91	113	6	2332	22	28

* Work of 2008, where the cutoff values were established [4].

**Table 2:** Reference strains of all genes. The highlighted genotypes represent the reference for that genotype. The first 41 strains were taken from Matthijnssens et al. in 2008 [28] and the others from the RCWG homepage, accessed in July 2024 [29].

Reference Strains for Each Genotypes											
Strain Name	VP7	VP4	VP6	VP1	VP2	VP3	NSP1	NSP2	NSP3	NSP4	NSP5
RVA/Human-tc/USA/WaCS/1974/G1P[8]	**G1**	**P[8]**	**I1**	**R1**	**C1**	**M1**	**A1**	**N1**	**T1**	**E1**	**H1**
RVA/Human-tc/USA/DS-1/1976/G2P[4]	**G2**	**P[4]**	**I2**	**R2**	**C2**	**M2**	**A2**	**N2**	**T2**	**E2**	**H2**
RVA/Human-tc/JPN/AU-1/1982/G3P[9]	**G3**	P[9]	**I3**	**R3**	**C3**	**M3**	**A3**	**N3**	**T3**	**E3**	**H3**
RVA/Human-tc/GBR/ST3/1975/G4P2A[6]	**G4**	P[6]	I1	R1	C1	M1	A1	N1	T1	E1	H1
RVA/Pig-tc/USA/Gottfried/1983/G4P[6]	G4	P[6]	I1	R1	C1	M1	**A8**	N1	T1	E1	H1
RVA/Pig-tc/USA/OSU/1975/G5P[7]	**G5**	**P[7]**	I5	R1	C1	M1	A1	N1	T1	E1	H1
RVA/Cow-tc/USA/NCDV-Lincoln/1971/G6P[1]	**G6**	**P[1]**	I2	R2	C2	M2	-	N2	**T6**	E2	-
RVA/Human-wt/HUN/Hun5/1997/G6P[14]	G6	P[14]	I2	R2	C2	M2	**A11**	N2	T6	E2	H3
RVA/Turkey-tc/IRL/Ty-3/1979/G7P[35]	**G7**	P[35]	I4	R4	C4	M4	A16	**N4**	T4	E11	H14
RVA/Human-wt/COD/DRC86/2003/G8P[6]	**G8**	P[6]	I2	R2	C2	M2	A2	N2	T2	E2	H2
RVA/Human-tc/USA/WI61/1983/G9P1A[8]	**G9**	P[8]	I1	R1	C1	M1	A1	N1	T1	E1	H1
RVA/Human/IND/I321/1996/G10P[11]	**G10**	P[11]	I2	-	-	-	A1	N2	T1	E2	-
RVA/Human-wt/BGD/Dhaka6/2001/G11P[25]	**G11**	P[25]	I1	R1	C1	M1	A1	N1	T1	E1	H1
RVA/Human-tc/PHL/L26/1987/G12P[4]	**G12**	P[4]	I2	R2	C2	M2	A2	N1	T2	E2	H1
RVA/Human-tc/THA/T152/1998/G12P[9]	G12	P[9]	I3	R3	C3	M3	**A12**	N3	T3	E3	**H6**
RVA/Horse-tc/GBR/L338/1991/G13P[18]	**G13**	**P[18]**	I6	**R9**	**C9**	**M6**	**A6**	**N9**	**T12**	**E14**	**H11**
RVA/Horse-tc/USA/FI23/1981/G14P[12]	**G14**	**P[12]**	I2	R2	C2	M3	A10	N2	T3	E2	H7
RVA/Cow/IND/Hg18/XXXX/G15P[21]	**G15**	**P[21]**	-	-	-	-	-	-	-	E2	-
RVA/Mouse-tc/USA/EDIM/XXXX/G16P[16]	**G16**	**P[16]**	**I7**	R7	C7	M8	**A7**	N7	T10	**E7**	H9
RVA/Turkey-tc/IRL/Ty-1/1979/G17P[38]	**G17**	**P[38]**	I4	R4	C4	M4	A16	N4	T4	E4	H4
RVA/Pigeon-tc/PO-13/1983/G18P[17]	**G18**	**P[17]**	**I4**	**R4**	**C4**	**M4**	**A4**	**N4**	**T4**	**E4**	**H4**
RVA/Chicken-tc/DEU/06V0661/2006/G19P[31]	**G19**	**P[31]**	I11	-	-	-	-	-	-	-	H8
RVA/Chicken-tc/IRL/Ch-1/1979/G19P[30]	G19	P[17]	**I10**	-	-	-	-	-	-	-	H10
RVA/Simian-tc/ZAF/SA11-H96/1958/G3P[2]	G3	**P[2]**	I2	R2	**C5**	**M5**	**A5**	**N5**	**T5**	E2	**H5**
RVA/Cat-tc/AUS/Cat97/1984/G3P[3]	G3	**P[3]**	I3	R3	C2	M3	A9	N2	T3	E3	**H6**
RVA/Cow-tc/USA/WC3/1981/G6P[5]	G6	**P[5]**	I2	R2	C2	M2	A3	N2	T6	E2	H3
RVA/Human-tc/GBR/ST3/1975/G4P2A[6]	G4	**P[6]**	I1	R1	C1	M1	A1	N1	T1	E1	H1
RVA/Cat-tc/AUS/Cat2/1984/G3P[9]	G3	**P[9]**	I3	R3	C2	M3	A3	N1	T6	E3	H3
RVA/Human-tc/IDN/69M/1980/G8P4[10]	G8	**P[10]**	I2	R2	C2	M2	A2	N2	T2	E2	H2
RVA/Cow-tc/THA/A5-13/1988/G8P[1]	G8	P[1]	-	-	-	-	**A14**	-	-	-	-
RVA/Cow-tc/USA/B223/1983/G10P[11]	G10	**P[11]**	I2	R2	C2	M2	**A13**	N2	T6	E2	H3
RVA/Horse-tc/GBR/H-2/1976/G3P[12]	G3	**P[12]**	**I6**	R2	C2	M3	**A10**	N2	T3	E2	H7
RVA/Human-wt/IND/HP140/1987/G6P[13]	G6	**P[13]**	I2	-	-	-	-	-	-	E1	H1
RVA/Sheep-tc/ESP/OVR762/2002/G8P[14]	G8	**P[14]**	I2	R2	C2	M2	A11	N2	T6	E2	H3
RVA/Ovine/CHN/Lp14/1981/G10P[15]	G10	**P[15]**	I2	-	-	-	-	-	-	E2	H3
RVA/Human/IND/RMC321/1989/G9P[19]	G9	**P[19]**	I5	-	-	-	A1	N1	T1	E1	H1
RVA/Mouse/Brazil/EHP/1981/G16P[20]	G16	**P[20]**	-	-	-	-	A7	-	-	E7	-
RVA/Rabbit/ITA/160-01/2002/G3P[22]	G3	**P[22]**	-	-	-	-	-	-	-	**E5**	-
RVA/Pig-wt/ESP/34461-4/2003/G2P[23]	G2	**P[23]**	**I5**	-	-	-	-	-	-	E1	H1
RVA/Rhesus-wt/USA/TUCH/2002/G3P[24]	G3	**P[24]**	**I9**	R3	C3	M3	**A9**	N1	T3	E3	H6
RVA/Human-wt/NPL/KTM368/2004/G11P[25]	G11	**P[25]**	**I12**	R1	C1	M1	A1	N1	T1	E1	H1
RVA/Human-tc/ITA/PA260-97/1997/G3P[3]	G3	P[3]	I3	R3	C3	M3	**A15**	N2	T3	E3	H6
RVA/Cow-wt/ARG/B383/1998/G15P[11]	G15	P[11]	I2	**R5**	C2	M2	A13	N2	T6	E12	H3
RVA/Horse-wt/ARG/E30/1993/G3P[12]	G3	P[12]	I6	R2	C2	M3	A10	N2	T3	**E12**	**H7**
RVA/Pig/ITA/134/04-15/2003/G5P[26]	G5	**P[26]**	I5	-	-	-	-	-	-	E1	**-**
RVA/Dog-tc/ITA/RV198-95/1995/G3P[3]	G3	P[3]	-	-	-	-	-	-	-	**E8**	-
RVA/Pig/THA/CMP034/2000/G20P[27]	G2	**P[27]**	I5	-	-	-	-	-	-	**E9**	**H1**
RVA/Human-wt/ECU/Ecu534/2006/G20P[28]	**G20**	**P[28]**	**I13**	R13	C13	M12	A23	N13	T15	E20	H15
RVA/Cow-wt/JPN/Azuk-1/2006/G21P[29]	**G21**	**P[29]**	I2	R2	C2	M2	A13	N2	T9	E2	H3
RVA/Turkey-tc/DEU/03V0002E10/2003/G22P[35]	**G22**	**P[35]**	I4	R4	C4	M4	A16	N4	T4	E11	H4
RVA/Chicken-tc/DEU/02V0002G3/2002/G19P[30]	G19	**P[30]**	I11	**R6**	**C6**	**M7**	**A16**	**N6**	**T8**	E10	**H8**
RVA/Pig-wt/IRL/61-07-ire/2007/G2P[32]	G2	**P[32]**	-	-	-	-	-	-	-	-	-
RVA/Pheasant-wt/HUN/Phea14246/2008/G23P[x]	**G23**	-	-	-	-	-	-	-	-	-	-
RVA/Pig-wt/CAN/CE-M-06-0003/2005/G2P[27]	G2	P[27]	**I14**	-	-	-	-	-	-	-	-
RVA/Cow-tc/JPN/Dai-10/2007/G24P[33]	**G24**	**P[33]**	I2	R2	C2	M2	A13	N2	T9	E2	H3
RVA/Cow-wt/JPN/Azuk-1/2006/G21P[29]	G21	P[29]	I2	R2	C2	M2	A13	N2	**T9**	E2	H3
RVA/Mouse-tc/USA/ETD_822/XXXX/G16P[16]	G16	P[16]	I7	**R7**	**C7**	**M8**	A7	**N7**	**T10**	E7	**H9**
RVA/Bat-wt/KEN/KE4852/07/2007/G25P[6]	**G25**	P[6]	**I15**	-	**C8**	-	-	**N8**	**T11**	E2	**H10**
RVA/Pig-wt/JPN/FGP51/2009/G4P[34]	G4	**P[34]**	-	-	-	-	-	-	-	-	-
RVA/Human-tc/KEN/B10/1987/G3P[2]	G3	P[2]	**I16**	**R8**	C5	M5	A5	N5	T5	E13	H5
RVA/Pig-wt/JPN/TJ4-1/2010/G26P[X]	**G26**	-	-	-	-	-	-	-	-	-	-
RVA/SugarGlider-tc/JPN/SG385/2012/G27P[36]	**G27**	**P[36]**	**I19**	**R10**	**C10**	**M9**	**A20**	**N11**	**T13**	**E17**	**H12**
RVA/Camel-wt/KUW/21s/2010/G10P[15]	G10	P[15]	I1	R1	C2	-	-	N2	T2	**E15**	H3
RVA/vicugna-wt/ARG/C75/2010/G8P[14]	G8	P[14]	I2	R2	C2	M2	-	N2	T6	**E16**	-
RVA/Rabbit-tc/CHN/N5/1992/G3P[14]	G3	P[14]	**I17**	R3	C3	M3	A9	N1	T1	E3	H2
RVA/Alpaca-wt/PER/356/2010/G3P[14]	G3	P[14]	I2	R5	C3	M3	**A17**	N3	T6	E3	H3
RVA/Pheasant-tc/GER/10V0112H5/2010/G23P[37]	G23	**P[37]**	I4	R4	C4	M4	A16	**N10**	T4	E4	H4
RVA/Camel-wt/SDN/MRC-DPRU447/2004/G8P[11]	G8	P[11]	I2	R2	C2	M2	**A18**	N2	T6	E2	H3
RVA/Human-wt/BRA/QUI-35-F5/2010/G3P[9]	G3	P[9]	**I18**	R3	C3	M3	**A19**	N3	T3	E3	H6
RVA/VelvetScoter-tc/JPN/RK1/1989/G18P[17]	G18	P[17]	I4	R4	C4	M4	**A21**	N4	T4	E4	H4
RVA/Rat-wt/GER/KS-11-573/2011/G3P[3]	G3	P[3]	**I20**	**R11**	**C11**	**M10**	**A22**	N2	**T14**	**E18**	**H13**
RVA/Fox-wt/ITA/288356/2011/G18P[17]	G18	P[17]	I4	R4	C4	M4	A16	N4	T4	**E19**	H4
RVA/Turkey-tc/IRL/Ty-3/1979/G7P[17]	G7	P[17]	I4	R4	C4	M4	A16	N4	T4	E11	**H14**
RVA/Human-wt/ITA/ME848-12/2012/G12P[8]	G12	P[8]	I17	**R12**	**C12**	**M11**	A12	**N12**	**T7**	**E6**	H2
RVA/Human-wt/SUR/2014735512/2013/G20P[28]	G20	P[28]		**R13**	**C13**	**M12**	**A23**	**N13**	**T15**	**E20**	**H15**
RVA/Common_Gull-wt/JPN/Ho374/2013/G28P[39]	**G28**	**P[39]**	**I21**	**R14**	**C14**	**M13**	**A24**	**N14**	**T16**	**E21**	**H16**
RVA/Alpaca-tc/PER/SA44/2014/G3P[40]	G3	**P[40]**	**I8**	R3	C3	M3	A9	N3	T3	E3	H6
RVA/Human-wt/BEL/BEF06018/2014/G29P[41]	**G29**	**P[41]**	I2	R2	C2	M2	A3	N2	T6	E2	H3
Strain name	VP7	VP4	VP6	VP1	VP2	VP3	NSP1	NSP2	NSP3	NSP4	NSP5
RVA/Bat-wt/CMR/BatLi09/2014/G30P[42]	**G30**	**P[42]**	**I22**	**R15**	**C15**	M14	**A25**	**N15**	**T17**	**E22**	**H17**
RVA/Bat-wt/CMR/BatLi08/2014/G31P[42]	**G31**	P[42]	I22	R15	C15	M14	A25	N15	T17	E22	H17
RVA/Bat-wt/CMR/BatLi10/2014/G30P[42]	G30	P[42]	I23	R15	C15	**M14**	A25	N15	T17	E22	H17
RVA/Bat-wt/CMR/BatLy03/2014/G25P[43]	G25	**P[43]**	I15	**R16**	C8	**M15**	**A26**	N8	T11	**E23**	H10
RVA/Bat-wt/NLD/NPpipi1/2014/GxP[44]	-	**P[44]**	**I23**	**R17**	**C16**	**M16**	**A27**	**N16**	**T18**	-	**H18**
RVA/Rat-wt/CHN/RA116/2013/G3P[45]	G3	**P[45]**	I3	R3	C3	M10	A22	N3	T3	E3	H13
RVA/Shrew-wt/CHN/LW9/2013/G32P[46]	**G32**	**P[46]**	**I24**	**R18**	**C17**	**M17**	**A28**	**N17**	**T19**	**E24**	**H19**
RVA/Bat-wt/CMR/BatLy17/2014/G30P[47]	G30	**P[47]**	I22	R15	C15	M14	A25	N15	T17	E22	H17
RVA/Rat-wt/ITA/Rat14/2015/G3P[3]	G3	P[3]	I1	R11	C11	M10	A22	**N18**	T14	E18	H13
RVA/Bat-wt/CHN/GLRL1/2005/G33P[48]	**G33**	**P[48]**	**I25**	**R19**	**C18**	**M18**	-	**N19**	**T20**	**E25**	**H20**
RVA/Bat-wt/CHN/YSSK5/2015/G3P[3]	G3	P[3]	I8	**R20**	C2	M1	A9	N3	T3	E3	H6
RVA/Bat-wt/CHN/BSTM70/2015/G3P[3]	G3	P[3]	I8	R3	C3	M3	**A29**	N3	T3	E3	H6
RVA/Raccoon-wt/JPN/Rac-311/2011/G34P[17]	**G34**	P[17]	**I26**	**R21**	**C19**	**M19**	**A30**	**N20**	**T21**	**E26**	**H21**
RVA/Pig-wt/BGD/214016006/2014/G9P[49]	G9	**P[49]**	-	-	-	-	-	-	-	-	-
RVA/Alpaca-wt/PER/Alp11B/2010/G35P[50]	**G35**	**P[50]**	I13	-	-	-	-	-	-	E16	H6
RVA/Bat-wt/ZMB/ZFB14-126/2014/GxP[x]	-	-	I22	-	-	-	-	**N21**	T17	**E27**	-
RVA/Bat-wt/KEN/BATp39/2015/G36P[51]	**G36**	**P[51]**	I16	**R22**	**C20**	**M20**	**A31**	**N22**	**T22**	E27	**H22**
RVA/Bat/CRC/KCR10-93/2010/G20P[47]	G20	P[47]	I13	R13	C13	M12	**A32**	N13	**T23**	E20	-
RVA/Bat/GAB/GKS-929/2009/G3P[2]	G3	P[2]	**I30**	R8	C5	M5	**A36**	**N23**	T5	**E28**	H5
RVA/Bovine-wt/UMN-VDL/2018/G37P[52]	**G37**	**P[52]**	-	-	-	-	-	-	-	-	-
RVA/common-shrew/KS-11-2281/2011/GXP[X]	-	-	**I27**	**R23**	-	-	-	-	-	-	**H23**
RVA/Bat-wt/KEN/11/2008/G30P[53]	G30	**P[53]**	-	-	-	-	**A33**	-	-	-	**H24**
RVA/Bat-wt/GTM/56/2010/G38P[54]	**G38**	**P[54]**	**I28**	**R24**	**C21**	**M21**	**A34**	**N24**	**T24**	**E29**	**H25**
RVA/Bat-wt/NGA/59/2011/GXP[2]	-	P[2]	I16	R8	C3	M5	**A35**	N3	T3	-	-
RVA/Bat-wt/GTM/53/2009/GxPx	-	-	**I29**	**R25**	-	-	-	**N25**	**T25**	-	-
RVA/Shrew-wt/GER/KS14-269/2014/G39P[55]	**G39**	**P[55]**	-	**R26**	**C22**	**M22**	**A37**	**N26**	**T26**	**E30**	**H26**
RVA/JungleCrow-wt/JPN/JC-105/2019/G40P[56]	**G40**	**P[56]**	I26	R21	C19	M19	A30	N20	T21	E26	H21
RVA/MultimammateMouse-wt/ZMB/MpR12/2012/G41P[57]	**G41**	**P[57]**	**I31**	**R27**	**C23**	**M23**	**A38**	**N27**	**T27**	**E31**	**H27**
RVA/Shrew-wt/GER/KS11-0893/2010/G42P[58]	**G42**	**P[58]**	**I32**	**R28**	**C24**	**L24**	**A39**	**N28**	**T28**	**E32**	**H28**

**Table 3:** Strains out of the cutoff value calculated with the *p*-distance model. Here, we compare the number of stains out of the cutoff value for each genotype, the number of strains out of the cutoff value for each gene, and the total of strains analyzed included in each particular gene.

Strains Out of the Cutoff Value with *p*-Distance Model				
Gene	Genotypes Outliers	Strains Outliers	Total Strains In Genotype	Rate
**VP7**	G3	1	384	0.3%
**VP4**	P[8]	10	1566	0.64%
	P[15]	1	14	7.1%
**VP6**	I2	13	773	1.7%
	I12	2	20	10.0%
**VP1**	none	-	1495	0%
**VP2**	C1	7	1789	0.4%
	C2	5	869	0.6%
**VP3**	None	-		0%
**NSP1**	None	-	2208	0%
**NSP2**	N1	14	1603	0.9%
	N2	6	644	0.9%
**NSP3**	T1	8	1496	0.5%
	T3	6	70	8.6%
**NSP4**	E1	5	1952	0.3%
	E2	56	881	6.4%
	E3	15	77	19.5%
**NSP5**	H1	3	1580	0.2%
	H2	2	514	0.4%
	H3	6	147	4.1%
	H10	5	5	100%
	H14	2	2	100%
	H15	2	2	100%
**TOTAL**		169		

**Table 4:** Compared results of the RVA strain cutoff analysis. We compared the number of sequences analyzed in 2008 with the two models used in this study. In the columns are the number of strains out of the cutoff value. In the case of 2008, the number of strains analyzed are also included.

Comparison Between Models				
Gene	2008 *	This Work		
	Strains Analyzed	Strains Analyzed	*p*-Distance Outside the Cutoff	K2P Outside the Cutoff
VP7	1000	3172	1	215
VP4	190	2771	11	45
VP6	142	2463	15	95
VP1	58	1495	-	262
VP2	58	2754	12	199
VP3	67	1507	-	165
NSP1	100	2208	-	502
NSP2	71	2358	20	1234
NSP3	77	2294	14	255
NSP4	100	3183	76	1025
NSP5	113	2332	20	252

* Work of 2008 [4].

## Data Availability

Data are contained within the article.

## Appendix A

**Table A1:** Base frequency and substitution rate of all sequences included in this study for each protein and the average values. The substitution rate for each pair of nucleotides was calculated. The evolution model used to construct the trees and the distance matrix. The gamma parameter used to correct the variation in the rate of substitution were estimated with bootstrap resampling with 1000 replicates to assess statistical robustness.

Base Frequency and Substitution Rate of All Genes and Evolution Models Suggested by MegaX													
Gene	Base Frequency %				Nucleotide Substitution Frequencies						Evolution Model *		Gamma Parameter
	G	A	T	C	G-T	G-A	G-C	A-T	A-C	T-C	Tree	Distance Matrix	
VP7	31	36	18	15	1	9.2	1.0	0.8	1.6	11.1	GTR + F + R8	Kimura 2-parameter	4
VP4	30	37	18	15	1	9.5	1.2	0.7	1.9	12.2	GTR + F + R7	Kimura 2-parameter	4
VP6	30	34	19	17	1	12.6	0.7	0.9	2.0	16.3	GTR + F + R5	Kimura 2-parameter	4
VP1	30	38	17	15	1	14.0	0.9	0.6	2.0	20.7	GTR + F + R10	Kimura 2-parameter	4
VP2	29	38	18	15	1	14.3	0.9	0.8	2.2	22.3	GTR + F + R8	Kimura 2-parameter	4
VP3	32	38	16	14	1	14.3	1.0	0.5	2.0	20.7	GTR + F + R10	Kimura 2-parameter	4
NSP1	37	17	33	13	1	10.0	1.1	0.5	2.1	14.4	GTR + F + R10	Tamura 3-parameter	0.88
NSP2	37	18	31	14	1	12.0	0.7	0.7	1.9	16.5	GTR + F + R10	Tamura 3-parameter	0.48
NSP3	38	19	31	12	1	10.9	1.0	0.6	2.0	17.4	GTR + F + R10	Tamura 3-parameter	0.56
NSP4	39	19	27	15	1	8.0	0.5	0.6	1.0	10.6	GTR + F + R10	Tamura-Nei	0.72
NSP5	35	19	29	17	1	8.8	0.8	0.9	1.6	10.4	GTR + F + R10	Tamura 3-parameter	0.51
Average	33.5	28.5	23.4	14.7	1.0	11.2	0.9	0.7	1.9	15.7			

* The evolutionary model used to construct the phylogenetic tree or the distance matrix.
